# Exercise, Erectile Dysfunction and Co-Morbidities: “The Good, the Bad and the Ugly”

**DOI:** 10.31083/j.rcm2309304

**Published:** 2022-09-09

**Authors:** Dimitris Patoulias, Alexandra Katsimardou, Konstantinos Imprialos, Michael Doumas

**Affiliations:** ^1^Second Propedeutic Department of Internal Medicine, Aristotle University of Thessaloniki, 54642 Thessaloniki, Greece

**Keywords:** erectile dysfunction, exercise, physical activity, cardiovascular disease, mortality

## Abstract

Erectile dysfunction (ED), defined as the inability to attain or maintain sufficient penile erection for sexual intercourse, is a growing health problem, which unfortunately remains underreported, underdiagnosed and undertreated. Growing evidence suggests that ED is a promising cardiovascular risk marker, as it is associated with major co-morbidities increasing cardiovascular disease burden, while it is an independent predictor of cardiovascular morbidity and mortality. The role of exercise as a non-pharmacological therapeutic intervention in ED has been widely investigated during the last two decades, both in observational studies and in randomized controlled trials, enrolling different patients’ populations. In the present narrative review, we summarize relevant evidence concerning the effect of exercise on vascular ED and the pathophysiologic background, underscoring the importance of enhanced physical activity as a recommendation in all subjects with vascular ED.

## 1. Erectile Dysfunction: An Increasing Health Burden

Erectile dysfunction (ED) is characterized by the inability to attain or maintain sufficient penile erection for achieving sexual intercourse [1]. Rapid assessment of the severity of ED in clinical practice is allowed with the use of the five-question International Index of Erectile Function (IIEF-5) [2].

Its prevalence is rising worldwide, mainly affecting older men, while it is estimated that 12 million men in the United States (US) aged 40–79 years old suffer from ED [2]. Results from the European Male Ageing Study (EMAS) in a sample of 3369 community-dwelling men aged 40–79 years old demonstrated that one out of three men reported ED, while 6% reported severe orgasmic impairment [3]. In the same study, the prevalence of moderate or severe ED was 6%, 19%, 38% and 64% among age groups 40–49, 50–59, 60–69 and above 70, respectively [3].

Formerly published data suggested an increasing prevalence of ED with age, lower education, and major co-morbidities, such as obesity, diabetes mellitus (DM), hypertension, metabolic syndrome and cardiovascular disease (CVD) [4, 5]. ED has both organic and psychogenic components, while, lifestyle factors, such as smoking, sedentary lifestyle and bicycle riding have also been implicated in increased risk for ED manifestation [6]. Moreover, several medications, such as antidepressants, antihypertensives, antiarrhythmics, diuretics, histamine receptor 2 antagonists, antiandrogens, corticosteroids, $5^{\alpha }$ reductase inhibitors, ketoconazole, and recreational drugs (alcohol, cannabis, cocaine), may also impair erectile function [6].

A meticulous medical and sexual history, a thorough physical examination with emphasis on the genitourinary tract, and routine hormonal and biochemical tests are required for an accurate diagnosis [7]. Physical examination should also focus on signs of hypogonadism, such as the presence of gynecomastia and the distribution of the body hair, and on the cardiovascular system, through measurements of heart rate and blood pressure and the assessment of heart rhythm and peripheral pulses for signs of arterial hypoperfusion, while the assessment of waist circumference and body mass index are also of great significance [7]. Regarding the etiology of ED, simple guided questions can help differentiate psychogenic from organic causes, as a sudden onset, an intermittent course and a short duration of ED point towards psychogenic factors, while a gradual onset, a progressive course and a longer duration are more indicative of an organic etiology [1].

Overall, normal sex life is considered important for the male population, however, only a minority of them request help from specialized healthcare professionals [8]. On the other hand, ED is not an issue commonly addressed by physicians during medical appointments for various reasons. Lack of expertise and practice, downgrading the issue in favor of other health-related problems and reluctance of clinicians to address the problem because they feel uncomfortable with such a discussion with their patients are some of them [1, 2].

## 2. Erectile Dysfunction and Main Co-Morbidities

DM, both type 1 and type 2, has been strongly associated with increased risk for ED. A former meta-analysis in a total of 88,577 men demonstrated that more than half of men with DM (52.5%) suffer from ED, while men with DM compared to healthy controls featured increased odds for ED [odds ratio (OR) = 3.62; 95% confidence interval (CI); 2.53 to 5.16] [9]. The interconnection between type 2 DM and ED is so close, so that previous data have suggested that screening for ED can improve the diagnostic accuracy of well-established risk scores for the assessment of underdiagnosed type 2 DM [10]. According to recent data from the German Diabetes Study enrolling patients with recently diagnosed DM, men with severe insulin-resistant DM have the highest prevalence of ED, equal to 52%, while the relative frequency of ED is much lower in patients with severe autoimmune DM, equal to 7% [11]. In the same cohort, the prevalence of ED in men with severe insulin-deficient DM, mild obesity-related DM and mild age-related DM was 31%, 18% and 39%, respectively, highly indicative of the significant impact of both insulin resistance and insulin deficiency on normal erectile function [11].

Hypertension has also been recognized as an important risk factor for ED [12, 13]. A previous meta-analysis of observational studies in a total of 121,641 male subjects documented that hypertension is associated with significantly increased odds for the development of ED (OR = 1.74, 95% CI; 1.52 to 2.00), without important geographical disparities [14]. Other meta-analytic data have also confirmed the significantly increased odds for ED in a background of hypertension (OR = 1.84, 95% CI; 1.58 to 2.14) [15], making clear this association.

Chronic kidney disease (CKD) is another, well-described risk factor for ED. A recent meta-analysis of observational studies in a total of 5986 men showed an overall prevalence of ED of 76% among subjects with a CKD diagnosis, while hemodialysis and transplant patients had a lower prevalence of ED [16]. Other meta-analyses have come to similar conclusions regarding the interconnection between CKD and ED [17]. Interestingly, recent data on the prevalence of ED among end-stage renal disease (ESRD) patients are more detailed, showing a prevalence of 59% among renal transplant recipients, 79% among patients on hemodialysis, 71% among patients on peritoneal dialysis and 82% among patients with ESRD starting dialysis [18].

Data regarding the association between ED and dyslipidemia are rather scarce. The overall prevalence of ED among patients with dyslipidemia has been demonstrated to be approximately 12% [19]. This association can also be hypothesized by the significant improvement of erectile function, as quantified by the IIEF score, with the use of lipid-lowering drugs, mainly statins [20, 21], although their beneficial effect is not generally accepted.

Obstructive sleep apnea (OSA) syndrome is another well-known risk factor for ED. A small meta-analysis demonstrated that patients with OSA syndrome feature an increased risk for ED by 82%, compared to subjects without baseline OSA [risk ratio (RR) = 1.82, 95% CI; 1.12 to 2.97] [22]. Subsequently, a larger meta-analysis confirmed this highly significant, clinical observation [23].

Metabolic syndrome is an additional risk factor for ED; a former meta-analysis documented that, subjects with metabolic syndrome experience an increased risk for ED by 60% compared to controls (RR = 1.60, 95% CI; 1.27 to 2.02) [24], a finding that was further confirmed by a later meta-analysis, which also showed that the plasma glucose component of the metabolic syndrome has the greatest prognostic value for the development of ED [25].

Finally, the presence of obesity increases substantially the risk for the development of ED. Specifically, results from the Massachusetts Male Aging Study and the Health Professionals Followup Study cohort showed that obesity doubles the risk for ED [26, 27, 28]. Similar results were also depicted in the European Male Aging Study, in which a body mass index (BMI) above 30 ${\mathrm{kg/m}}^{2}$ and a waist circumference above 102 cm correlated with worse sexual activity [29]. Interestingly, weight loss through lifestyle interventions or bariatric surgery exerts a positive effect on erectile function, with improvements on the participants’ IIEF scores [30, 31].

## 3. Erectile Dysfunction and Cardiovascular Disease

A strong association between ED and cardiovascular disease has been established over the years since they share common pathophysiologic mechanisms [32]. ED is highly prevalent among men with cardiovascular disease [33], while ED is an independent risk factor for the development of cardiovascular disease.

ED usually precedes symptomatic cardiovascular disease [34], therefore its identification by healthcare physicians appears to be of utmost importance. A former meta-analysis of observational studies in a total of 36,744 enrolled subjects documented that ED increases the risk for cardiovascular disease by 48% (RR = 1.48, 95% CI; 1.25 to 1.74), for coronary artery disease by 46% (RR = 1.46, 95% CI; 1.31 to 1.63) and for stroke by 35% (RR = 1.35, 95% CI; 1.19 to 1.54) [35]. A significant increase in the risk for all-cause death by 19% was also shown (RR = 1.19, 95% CI; 1.05 to 1.34) [35]. Later meta-analyses confirmed this hazardous association, also highlighting that shorter duration of ED, concomitant DM and current smoking are significant contributors to cardiovascular disease development [36, 37]. Severe ED appears also to predict a higher risk for cardiovascular disease development, compared to milder disease [36].

Besides its strong correlation with surrogate cardiovascular endpoints, ED also correlates with subclinical cardiovascular disease, emphasizing the significance of meticulous cardiovascular risk assessment of patients presenting with ED [38]. More specifically, it has been previously shown that ED is associated with significantly impaired endothelial function, as quantified by flow-mediated dilation (FMD), while it also correlates with increased carotid intima-media thickness (cIMT) [38]. According to a recently published, observational study, ED also correlates with impaired arterial stiffness, with pulse wave velocity (PWV) values found to be increased across the different stages of ED severity, underscoring the association between ED and subclinical cardiovascular disease [39].

Overall, ED represents a valuable cardiovascular risk marker, although it is usually neglected in clinical practice [40].

## 4. Pathophysiologic Mechanisms Implicated into Erectile Dysfunction

Normally, erection is the result of smooth muscle cell relaxation in the penis, enabling the inflow of blood in the corposa cavernosum and thereby the compression of the subtunical venules, blocking the venous outflow. Any interruption of this veno-occlusive mechanism can lead to the development of ED [6].

Endothelial dysfunction appears to play a catalytic role in the development of ED, with endothelium-derived nitric oxide (NO), synthesized by L-arginine [41], being decisive in regulating smooth muscle tone, and thus, being crucial for penile erection, through relaxation of cavernosal smooth muscles and subsequent compression of the subtunical small veins [1]. Impaired NO synthesis or availability has been demonstrated as a significant factor contributing to endothelial dysfunction, and thus, to ED, while, several diseases and drug classes have been shown to affect the expression of endothelial NO synthase (eNOS), and thus, regulating NO levels [41].

Enhanced oxidative stress and hyperglycemia-induced advanced glycation end (AGE) products seem also to be crucial for the development of ED in patients with DM [42]. Both conditions lead to eNOS inhibition, macromolecular damage, endothelial cell apoptosis and vascular endothelial signaling inhibition, therefore resulting in cavernosal endothelial dysfunction, impairment of vasorelaxation of smooth muscle cells, and finally in manifestation of ED [42].

There is also evidence suggesting a prominent role of testosterone in the pathogenesis of ED. Previous animal studies have shown that androgen deprivation results in structural alterations in the corpus cavernosum and finally, in reduced intracavernosal pressure [43]. Testosterone has been shown to upregulate eNOS, while it downregulates the activity of RhoA-ROCK (Ras homolog gene family member A-Rho-associated, coiled coil containing protein kinase) pathway, which controls the sensitization of penile smooth muscle cells to calcium, and as a result, regulates penile relaxation [44].

Subclinical inflammation and atherosclerosis seem to be crucial for the development of ED, as well. Carotid and coronary artery atherosclerosis has been shown to be highly predictive of ED in human studies [45, 46]. In addition, a significant association between the presence of cavernosal atherosclerotic plaques and ED has been documented [47]. Increasing evidence also suggests the association between ED and inflammation, since increased levels of pro-inflammatory cytokines have been found in ED, along with decreased levels of anti-inflammatory cytokines [48, 49].

**Fig. 1.**
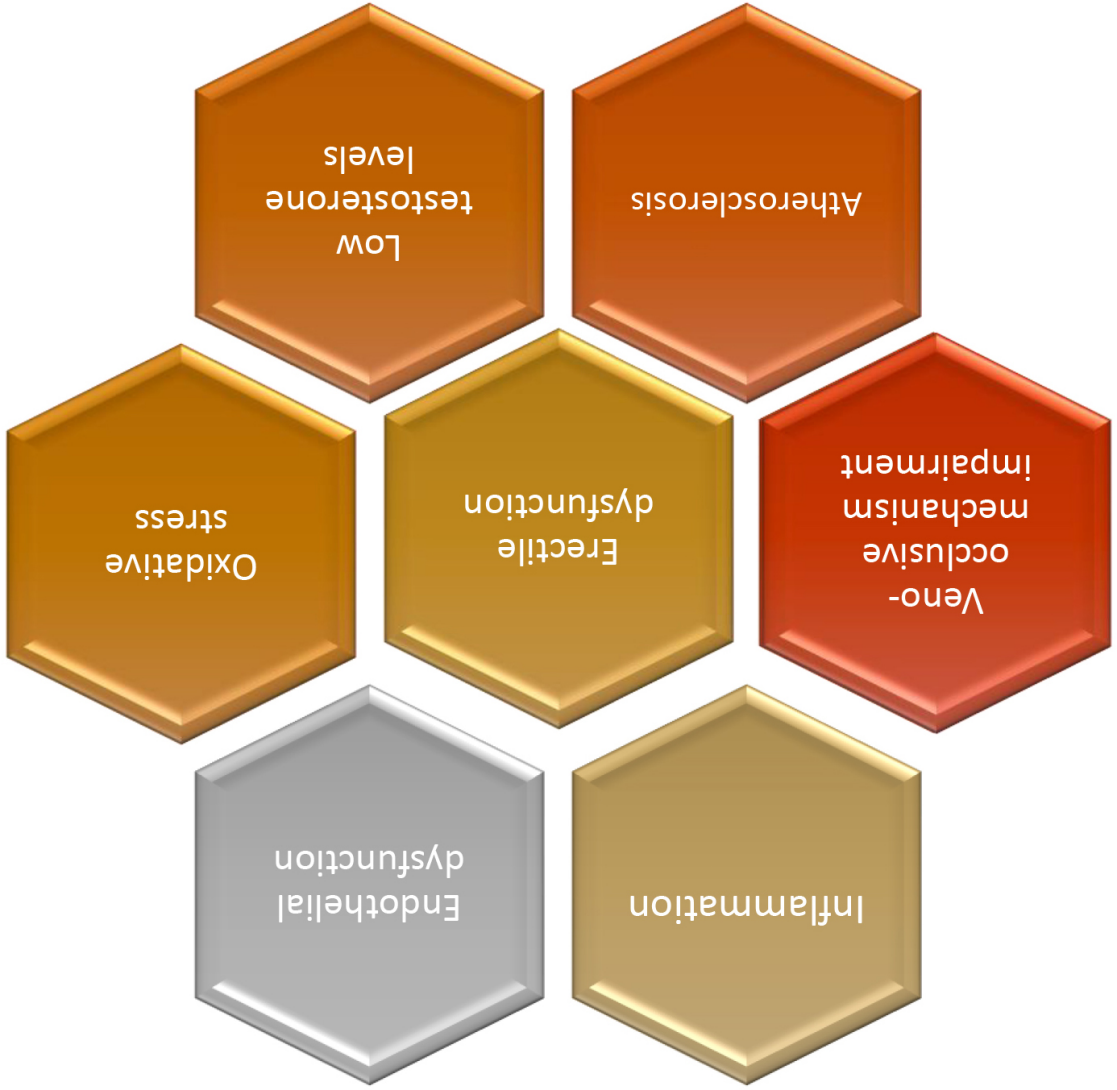
**Main pathophysiologic mechanisms implicated in the development of erectile dysfunction**.

Overall, it seems that there is a vicious circle between endothelial dysfunction, oxidative stress, atherosclerosis and inflammation leading to ED (Fig. 1), with NO representing the key mediator. However, a more comprehensive review of the main pathophysiologic mechanisms underlying ED is outside the scope of this manuscript and is discussed in detail elsewhere (for example, [1, 41, 42]).

## 5. Physical Activity and Erectile Dysfunction: Evidence from Observational Studies

A previous sub-analysis of the Look AHEAD trial (Action for Health in Diabetes), with a total of 373 men with type 2 DM demonstrated that 74.6% of them suffered from ED of different severity (29.1% from mild, 20.7% from moderate and 24.8% from severe ED), with 39.9% of them seeking for medical consultation, while 32.3% of them already used drugs with proven efficacy against ED [50]. Remarkably, cardiorespiratory fitness was significantly protective against ED [odds ratio (OR) = 0.61, 95% CI; 0.47 to 0.78, for each unit of fitness z-score], as shown in the multivariate analysis [50].

In another study from the field of cardiac rehabilitation, enrolling 138 men with confirmed ischemic heart disease and ED, it was shown that a 6-month exercise training program, including interval endurance training on a cycle ergometer, indoor or outdoor general fitness exercises and resistance training, resulted in a significant improvement in achieved erection quality (*p *< 0.05) [51]. A strong association between erection quality and exercise capacity was also shown [51]. A post-hoc analysis of this study revealed the significant correlation between the change in heart rate recovery and the change in erection quality among subjects following the intensive cardiac rehabilitation program, a finding highly suggestive of the role of autonomic dysfunction in ED [52].

Additional insights were provided by another observational study, enrolling 57 subjects with metabolic syndrome and 48 physically active controls [53]. Regular physical exercise at a level of >400 kcal/day was documented to be highly protective against ED (OR = 0.12, 95% CI; 0.017 to 0.778), while increased fibrinogen levels were shown to be predictive of the presence of ED [53]. In addition, large arterial elasticity was shown to be significantly lower among patients with metabolic syndrome compared to controls, and among patients with ED compared to those with normal erectile function [53]. Collectively, that study suggested once again the role of low-grade inflammation in ED, the vascular dysfunction observed in patients with ED and the protective role of physical activity against the occurrence of ED.

In a large, cross-sectional study from Finland, enrolling 1000 apparently healthy men, free from cardiovascular disease at baseline, it was shown that high-intensity physical activity is protective against ED, decreasing the corresponding odds by almost 50% (OR = 0.50, 95% CI; 0.29 to 0.86) [54]. However, lower intensity of exercise was not shown to ameliorate the risk for ED, highlighting once again the need for regular physical activity to maintain a normal erectile function [54].

## 6. Physical Activity and Erectile Dysfunction: Evidence from Randomized Controlled Trials

There has been a long and vivid discussion regarding the role of physical activity in ED prevention, primary or secondary.

A former randomized controlled trial (RCT) in a total of 110 obese men aged between 35 and 55 years old without baseline DM, hypertension, or dyslipidemia, demonstrated that a 2-year lifestyle intervention resulted in a significant improvement in erectile function [30]. Specifically, in the intervention arm, an increase in duration of physical activity, mainly aerobic exercise, from 48 to 195 minutes per week, led to a significant increase in IIEF score from 13.9 to 17 (*p *< 0.001), corresponding to a between-group difference of 3 (*p* = 0.008) with the control group [30]. Of note, physical activity was shown to be an independent predictor of IIEF score (*p* = 0.02) [30], highlighting its role in ED.

In another RCT enrolling a total of 60 patients with ED, with a mean age of 50 years and a mean BMI of 27 ${\mathrm{kg/m}}^{2}$, it was shown that recommendation of physical activity as an additional therapeutic intervention to phosphodiesterase type 5 inhibitor (PDE5i) treatment, compared to PDE5i treatment alone, resulted after 3 months in significant improvement in erectile function (*p* = 0.003), sexual desire (*p* = 0.028), intercourse satisfaction (*p* = 0.001) and IIEF score (*p* = 0.007) [55]. It is remarkable that physical activity greater than 180 minutes per week was the only independent predictor of normal erection (*p* = 0.01) and normal IIEF score (*p* = 0.023) [55]. In another trial enrolling a total of 50 subjects with ED, with a mean age of 57 years, without cardiovascular disease or DM at baseline, it was demonstrated that 150 minutes of moderate intensity aerobic activity per week resulted in a significant improvement in IIEF score after 3 months, compared to the control group (*p *< 0.05) [56]. Of interest, penile Doppler ultrasonographic assessment revealed a significant improvement in penile vascular markers, such as peak systolic velocity, acceleration time, end-diastolic velocity, resistance index and intimal cavernous thickness, in the physical activity group compared to the control group (*p *< 0.05 for all comparisons) [56]. Serum concentrations of endothelial progenitor cells and endothelial microparticles, indicative of endothelial apoptosis, were significantly lower in the physical activity group compared to the control group, as well [56].

Another RCT enrolling 75 obese, Asian men, with a mean age of 43.6 years, demonstrated that moderate intensity high-volume aerobic exercise of 200–300 minutes per week, compared to low-volume aerobic exercise of less than 150 minutes per week, resulted after a follow-up period of 24 weeks in a significant increase in IIEF score (*p *< 0.05), associated with a significant increase in plasma testosterone levels, highlighting the role of androgen deficiency in ED [57]. In another small trial recruiting 20 male patients with ED and metabolic syndrome, with a mean age of 68.5 years, it was once again shown that the combination of tadalafil treatment with a structured exercise training program (3 exercise sessions per week, each session was performed for 30 minutes on a bicycle ergometer or a treadmill) compared to tadalafil treatment alone resulted in a significant increase in IIEF score achieved after 2 months of the applied therapeutic intervention (*p *< 0.001), while a significant positive association between exercise capacity and erectile function in the whole study cohort was also documented [58].

A formerly published RCT enrolling 85 patients with a recent acute myocardial infarction and ED, who were randomized either to a home-based walking program followed by an outdoor progressive walking program or standard of care, showed that after one month, the exercise group experienced a significant improvement in erectile function (*p *< 0.0001), while, a significant negative correlation between the presence of ED and the 6-minute walk test (6-MWT) at 30 days after hospital discharge was also documented (*p *< 0.01) [59]. Another trial recruiting 83 patients with ED in the presence of type 2 DM showed that 3-month intensive glycemic control along with advice for physical exercise was inferior to intensive glycemic control and treatment with sildenafil concerning erectile function, as assessed with IIEF score, since patients allocated to sildenafil treatment experienced a greater improvement compared to those allocated to exercise group (*p* = 0.012) [60]. Of note, in the entire cohort, patients with a duration of DM less than 5 years experienced a greater improvement in erectile function, compared to those having DM more than 10 years [60].

In a pilot trial enrolling 50 patients with ED and testosterone deficiency, it was shown that a combination of a supervised 20-week physical activity program (3 times per week, 80 minutes in total in each session, consisting of 20 minutes of aerobic exercise, followed by 10 minutes of whole-body stretching and 30 minutes of strength exercise with the last 20 minutes consisting of aerobic exercise once again) with testosterone replacement therapy, compared to testosterone replacement therapy alone, led to a more pronounced improvement in IIEF score (*p* = 0.028), along with a greater increase in serum testosterone levels (*p *< 0.001) [61]. What is more, these improvements were maintained after the discontinuation of testosterone replacement in the exercise group, emphasizing its prominent role in ED [61].

A former RCT in a total of 154 patients with ischemic heart disease or implantable cardioverter defibrillator and ED showed that a 12-week combined intervention, including physical exercise training (3 weekly sessions, each one lasting 60 minutes, either supervised or home-based, including bicycling, strength training and stretching exercises), pelvic floor exercise and psychoeducation, compared to usual care, resulted in a significant increase in IIEF score (*p *< 0.0003), which persisted at 6 months post intervention [62]. Orgasmic function, sexual desire and intercourse satisfaction were also significantly improved in the physical exercise treatment arm [62].

Another RCT enrolling 50 men with localized prostate adenocarcinoma, stage I-II, and ED, demonstrated that a 6-month intervention with an aerobic exercise program (5 supervised walking sessions per week, 30–45 minutes per session, at 55–100% of ${\mathrm{VO}}_{2}$ peak), compared to usual care, led to a significant increase in IIEF score (*p* = 0.002), along with a significant improvement in orgasmic satisfaction, sexual desire and intercourse satisfaction; however, no significant between-group difference across prespecified outcomes was shown [63]. Of note, exercise resulted in a significant improvement in endothelial function, as assessed with FMD [63].

Implementation of an interval exercise training program, with the use of a bicycle ergometer at a low intensity of between 60% and 79% of maximum heart rate reserve, in subjects with hypertension and ED for 8 weeks, has been previously shown to improve their erectile function compared to standard of care (*p *< 0.05) [64]. A significant negative correlation between ED and C-reactive protein (CRP) was also shown, further highlighting the role of subclinical low-grade inflammation in the pathogenesis of ED [64].

A small, recently published RCT enrolling 37 patients undergoing hemodialysis (26 men in total) documented that those patients allocated to the intervention group, consisting of passive pedaling with the use of automatic mini bikes in each hemodialysis session for 20 minutes at a steady rate during the first 2 hours of dialysis, did not result after 12 weeks of intervention in a significant improvement in sexual function [65]. Therefore, passive forms of exercise might not be indicated for ED.

Overall, evidence retrieved from the limited, relevant RCTs suggests that physical exercise, especially aerobic, is associated with a significant improvement in erectile function among men with ED with or without cardiovascular risk factors or established cardiovascular disease. The nature of the intervention in the before mentioned RCTs is also something to consider. Patients were not only advised to exercise regularly and adopt healthier diet schedules, but most importantly, they were followed closely either through monthly visits, telephone calls, small group sessions or supervised exercise programs throughout the intervention period.

## 7. Physical Activity and Erectile Dysfunction: Evidence from Systematic Reviews and Meta-Analysis

Due to the diversity of the patients included in the RCTs and the interventions regarding physical activity, not many meta-analyses have been done so far regarding this matter. A meta-analysis that included seven eligible studies showed that physical exercise exerts a statistically significant improvement in erectile function. Specifically, a 3.85-point difference was observed in erectile function scores after the implementation of physical activity (95% CI; 2.33 to 5.37), with both short-term and long-term interventions being effective. In subgroup analysis erectile function scores were significantly improved in patients with cardiovascular risk factors alone (4.20; 95% CI; 2.16 to 6.23) and those with either coronary heart disease or radical prostatectomy (2.11; 95% CI; 0.76 to 3.45). However, the predisposing factors for erectile dysfunction, the type and duration of activity and the use of pharmacological treatment differed among the included studies [66].

Moreover, Gerbild *et al*. [67] conducted a systematic review that sought to determine the levels and type of physical activity needed to improve erectile dysfunction. The study was focused on patients with physical inactivity, obesity, hypertension, metabolic syndrome and cardiovascular disease. The authors stated that a meta-analysis could not be performed, as there was significant heterogeneity in the methods used among the included studies. Specifically, the included population, the assessment of erectile dysfunction and the type, duration, intensity and frequency of physical exercise differed to a great extent among the included studies. Nevertheless, the beneficial effects of physical activity on erectile dysfunction were evident, and the authors concluded that supervised aerobic physical activity of moderate to vigorous intensity performed at least four times per week and for at least 40 minutes can be a valuable and effective tool in the management of erectile dysfunction [67].

## 8. Physical Activity, Cardiovascular and All-Cause Mortality

Cardiorespiratory fitness has been shown to be a potential predictor of cardiovascular and all-cause mortality, both in the general population and in patients with a previous history of cardiovascular disease.

A former meta-analysis in a total of 102,980 healthy participants confirmed that per 1-metabolic equivalent (MET) higher level of maximal aerobic capacity (MAC) the corresponding risk for all-cause mortality was reduced by 13% (RR = 0.87, 95% CI; 0.84 to 0.90) and the risk for cardiovascular disease was reduced by 15% (RR = 0.85, 95% CI; 0.82 to 0.88) [68]. Patients with low cardiorespiratory fitness had a significantly increased risk for all-cause mortality by 70% and for a major adverse cardiovascular event by 56%, emphasizing the value of physical activity in the field of primary prevention of cardiovascular disease [68].

Among subjects with prior cardiovascular disease, it has also been confirmed by a recent meta-analysis in a total of 159,352 patients that the risk for all-cause mortality was reduced by 19% for each increase in MAC by 1 MET [hazard ratio (HR) = 0.81, 95% CI; 0.74 to 0.88]; however, no statistically significant association between cardiorespiratory fitness and cardiovascular mortality was shown (HR = 0.75, 95% CI; 0.48 to 1.18) [69]. Of note, an increase in MAC by 1 MET among patients with a history of coronary artery disease was shown to decrease the risk for all-cause mortality by 17% (HR = 0.83, 95% CI; 0.76 to 0.91); however, this benefit was not statistically confirmed for patients with a history of heart failure (HR = 0.69, 95% CI; 0.36 to 1.32) [69].

Overall, any level of physical activity has been shown to decrease the risk for all-cause death, as opposed to a sedentary lifestyle [70]. Interestingly, in terms of secondary prevention, exercise-based interventions seem to be similarly efficacious to drug-based interventions for patients with coronary artery disease, stroke or heart failure [71], underscoring the significance of physical exercise and its incorporation in cardiac rehabilitation programs [72].

Based upon the significantly increased prevalence of ED among these populations, as discussed in a previous section, it seems that, besides improvement in ED, the implementation of physical exercise programs can result in a significant decrease in cardiovascular and all-cause mortality.

## 9. Physical Activity and Pathophysiologic Mechanisms Implicated into Erectile Dysfunction

Exercise has been associated with a significant improvement in markers of inflammation, such as CRP, fibrinogen and pro-inflammatory cytokines, especially in patients with established atherosclerotic cardiovascular disease [73, 74]. However, it is not clear whether high-intensity activity produces a greater reduction in inflammatory burden, as demonstrated in a former meta-analysis [75].

In addition, exercise has been associated with a significant reduction in pro-oxidant products and an increase in antioxidant capacity, confirming the rationale for the antioxidant effects of exercise [76]. In specific patient populations, such as those with concomitant heart failure, exercise may have incremental antioxidant efficacy [77].

All exercise modalities (aerobic, resistance, combined) have been documented to exert a beneficial effect on endothelial function [78]. Regarding resistance training, it has been shown that it produces a significant improvement in endothelial function, regardless of a history of cardiovascular or chronic metabolic disease [79]. Low- to moderate-intensity resistance training program seems to be more efficacious in improving endothelial function, compared to high-intensity [80]. Improvement in endothelial function may be more pronounced in patients with type 2 DM compared to non-diabetic subjects [81].

Exercise also improves macrovascular dysfunction indices, such as arterial stiffness [82]. Of note, aerobic exercise provides a greater reduction in pulse wave velocity, the “gold-standard” of arterial stiffness, compared to other forms of exercise [83, 84]. However, all exercise training interventions have a beneficial effect on arterial stiffness indices [85].

Thus, it appears that exercise ameliorates the major pathophysiologic mechanisms implicated in the pathogenesis of ED.

## 10. Phosphodiesterase-5 Inhibitors and Exercise Capacity

PDE-5 inhibitors are recommended as the first-line treatment option in ED, with sildenafil being the first member of the class, followed by tadalafil, vardenafil, and avanafil [86, 87]. Combination with other drug classes or treatment approaches, such as antioxidants, low-intensity shockwave therapy, vacuum erectile device, folic acid, metformin hydrochloride, or angiotensin-converting enzyme inhibitors, may be considered in refractory or complicated cases [87].

Sildenafil treatment failed to produce a significant benefit on exercise capacity among patients with heart failure with preserved ejection fraction after 24 weeks of treatment, as demonstrated in the RELAX trial published in 2013 [88]. On the other hand, PDE-5 inhibitors have been shown to improve exercise capacity in patients with heart failure with reduced ejection fraction, as documented by relevant RCTs [89, 90]. These results have been also confirmed by relevant meta-analyses in the field [91, 92].

Other patient populations, such as those suffering from chronic obstructive pulmonary disease, did not also have any remarkable benefit concerning exercise capacity with PDE-5 inhibitor treatment [93]. No benefit on exercise capacity has been shown with PDE-5 inhibition in other chronic respiratory failure disorders, such as idiopathic pulmonary fibrosis [94].

Therefore, at present, it does not seem reasonable to support the hypothesis that treatment with PDE-5 inhibitors can improve exercise capacity in patients with ED, and thus can lead to additional improvement in erectile function.

## 11. Practical Recommendations Regarding Physical Activity in Patients with Erectile Dysfunction

Apart from the positive psychological effects, physical activity is a valuable tool for overall health improvement. For that reason, the World Health Organization has proposed in detail the amount and type of physical exercise necessary for every age group. Specifically, adults aged 18 to 65 years old should exercise at least 150 to 300 minutes per week, preferably with moderate intensity aerobic exercise. Muscle-strengthening activities should also be performed on two or more days per week [95]. Similarly, a recent meta-analysis showed that 40 minutes for four times per week and for six months of aerobic exercise of moderate intensity combined with resistance training improves significantly the erectile function of males with arterial ED and relevant comorbidities [67]. However, up to date no specific guidelines exist that can guide clinicians and patients in detail about the duration, the intensity and the type of exercise.

It appears that a mixture of different kinds of activities is more beneficial for patients. Aerobic activity, resistance training, combat sports and group activities help patients in various ways, suggesting that a plan combining these training modalities should be encouraged in patients with ED [96]. In a recent review Allen concluded that a combination of a high intensity whole body resistance training two times per week with a moderate intensity aerobic exercise for two days per week and a group activity one day per week with a minimum duration of 45 minutes per session and for more than 16 weeks can be a practical suggestion for such patients. Also, as low adherence to such suggestions is a very common problem in every day clinical practice, of utmost importance is the adoption of strategies to maximize adherence, such as supervised training programs, the adoption of training diaries, the provision of simple instructions and the continuous education of the patients about the benefits of exercise not only on ED, but also in health overall [96].

## 12. Conclusions

ED represents a growing health problem, significantly affecting patients’ quality of life, although it remains underreported, and thus underdiagnosed and undertreated. It has been established over the last 2 decades as a marker of cardiovascular disease, even at the subclinical level, while it seems to be highly prevalent among subjects with other cardiovascular risk factors.

Exercise represents an intervention that can provide substantial improvement in erectile function of the affected subjects, since it improves most of the implicated pathophysiologic mechanisms. It is therefore of utmost importance to recommend various forms of physical activity, according to the subject’s preferences and after meticulous assessment of co-morbidities, exercise capacity and other demographic factors of specific interest, in patients with ED. Such an approach can result not only in improvement in ED parameters, but also in the overall cardiovascular risk of the patient.
